# Right heart in septic shock: prospective observational study

**DOI:** 10.1186/s40560-016-0159-y

**Published:** 2016-06-07

**Authors:** Ratender Kumar Singh, Sudeep Kumar, Sreevatsa Nadig, Arvind Kumar Baronia, Banani Poddar, Afzal Azim, Mohan Gurjar

**Affiliations:** Department of Critical Care Medicine, Sanjay Gandhi Post Graduate Institute of Medical Sciences, Lucknow, Uttar Pradesh 226014 India; Department of Cardiology, Sanjay Gandhi Post Graduate Institute of Medical Sciences, Lucknow, Uttar Pradesh 226014 India

**Keywords:** Right heart, Septic shock, Transthoracic echocardiography

## Abstract

**Background:**

The right heart often receives less attention during echocardiography. The situation is no different in septic shock. We prospectively investigated the echocardiographic indices of the right heart in septic shock adult patients.

**Methods:**

Septic shock ICU patients within 24 h of admission were subjected to transthoracic echocardiography (TTE) as per the 2005 guidelines from the American Society of Echocardiography.

**Results:**

Eighty-eight septic shock patients (M:F = 52:36) underwent TTE. Thirty-six patients survived. Significant differences in demographic and biochemical (laboratory and metabolic) parameters, severity scores, life-support therapies (vasopressors, ventilation), and length of ICU stay were observed between survivors and non-survivors. Right heart abnormalities of chamber dimension and systolic and diastolic function existed in 79, 25, and 86 % of patients, respectively. Right ventricle subcostal wall thickness (91 %), pulse Doppler myocardial performance index (73 %), and E/E′ (63 %) were the predominant abnormalities in chamber dimension, systolic function, and diastolic function of the right heart, respectively. However, the presence of these abnormalities did not signify poor survival in our study.

**Conclusions:**

Right heart dimensional and functional abnormalities exist in high proportions in septic shock. However, their predictability of poor outcomes remains questionable.

## Background

Complex geometry, ill-defined endocardial surface, and retrosternal right ventricle (RV) impose challenges to imaging of the right heart (RH) [1]. This has often resulted in RH receiving less attention during echocardiography. Unfamiliarity with optimal ultrasound imaging techniques and absence of RH data indexed to gender, height, and weight have only widened the gap. Both transesophageal echocardiography (TEE) [2–5] and transthoracic echocardiography (TTE) [6–10] have been used to study the dimensional and functional aspects of RH. Easy and ready availability of TTE has made it more popular than TEE in most ICUs. Sepsis is a clinical syndrome caused by severe infection and is characterized by a systemic inflammation response syndrome with varying degrees of organ dysfunction [11]. The entire cardiovascular system is involved in sepsis and septic shock [12]. However, despite three decades of recognition of sepsis-associated myocardial dysfunction [13], the RH has been studied only sparingly. In a meta-analysis on severe sepsis and septic shock, only six studies out of a total of 14 have RH in focus [14]. Amongst these, only two [7, 8] have used TTE. Furthermore, the impact of RH dysfunction during septic shock is not clear. The absence of a universally accepted definition of RH dysfunction has only compounded the issue.

Systematic comprehensive quantitative approach to assessment of RH by TTE was given by the American Society of Echocardiography (ASE) and endorsed by the European and Canadian Society of Echocardiography [15]. Based on this approach, we endeavored to explore the impact of RH function on management and outcomes in septic shock.

## Methods

### Baseline clinical characteristics of the study population

The period of our study was from 2012 to 2014. It commenced after prior approval from Sanjay Gandhi Post Graduate Institute of Medical Sciences, ethics committee. Prior written informed consent was taken from the patient or next of kin as appropriate. The study included consecutive septic shock adult patients (≥18 years) admitted to the ICU of a tertiary care referral hospital and academic institute in north of India. Septic shock was defined as sepsis with hypotension despite initial volume resuscitation in accordance with the American College of Chest physicians (ACCP)/Society of Critical Care Medicine (SCCM) Consensus Conference Committee [11].

Relevant demographics [age (years), gender (M:F)]; type of illness [medical (lung-pneumonia, bronchial asthma, chronic obstructive pulmonary disease), kidney (acute kidney injury, chronic kidney disease, or acute on chronic kidney disease), or surgical (postoperative)]; severity scores [acute physiology and chronic health evaluation (APACHE-II) and sequential organ failure assessment (SOFA)]; laboratory variables; hemodynamic variables [heart rate, mean arterial pressure (MAP), central venous pressure (CVP) in mmHg, central venous oxygen saturation (CvO_2_ %) and lactate (mg/dl)]; acid-base variables [pH, base deficit or excess, partial pressure of arterial oxygen/carbon dioxide (PaO_2_/PaCO_2_), serum electrolytes {sodium (Na^+^), potassium (K^+^), chloride (Cl^−^)}], fluid balance (previous 24 h, positive/negative); urine output (UOP) [unaided, aided (diuretic induced or dialysis supported)] and relevant ICU-specific supportive therapeutic measures [vasopressors (type and dose), mechanical ventilation [pressure support (PS) and positive end-expiratory pressure (PEEP) cm H_2_O pressure, tidal volume (ml) and PaO_2_/FiO_2_ ratio]; and renal replacement therapy (RRT)] were recorded. Twenty-eight-day mortality and free days from event (vasopressor/ventilation/RRT) during ICU stay were also documented.

### Right heart transthoracic echocardiography

A bed side TTE using MicroMaxx Ultrasound System with P17/5-1 MHz cardiac probe was performed within the first 24 h of ICU admission in the post-resuscitation phase of septic shock. Optimal positioning for good echocardiographic window of the patient was ensured before conducting the TTE. The TTE data was recorded by a trained cardiologist from the department of cardiology of our institute. Dimensions averaged over couple of beats were noted. The RH indices were measured using motion (M)-mode, two-dimensional (2D) images, and Doppler (pulsed/tissue) as per ASE guidelines [15]. Left heart (LH) data was also simultaneously recorded.

### Right heart dimensional and functional indices

The chamber dimensions (mm) included: major and minor dimensions of the right atrium (RA); RA end-systolic area (ESA, cm^2^); RV-basal diameter; subcostal wall thickness; and parasternal short (distal diameter)-axis/long (proximal diameter)-axis (PSAX/PLAX) outflow tract. RV systolic function indices included: tricuspid annular plane systolic excursion (TAPSE); pulsed Doppler (PD) peak velocity at annulus; PD myocardial performance index (MPI); tissue Doppler (TD) MPI; and fractional area change (FAC, %). RV diastolic function indices included: E/A ratio, E/E′ ratio, and deceleration time (ms). The normal non-indexed reference values from non-ICU population [15] for the above measures are displayed in Table 2. RH TTE indices of dimension or function were further categorized into either normal or abnormal in relation to available reference normal values. LH indices of dimension and function were also measured and left ventricle (LV) systolic dysfunction was defined as LVEF < 50 % in our study.

### Statistical analysis

Statistical analysis was done with SPSS (SPSS Inc., Chicago, IL, USA) version 17 for Windows. Data was expressed as mean ± SD or absolute numbers or proportions as appropriate. Continuous data was compared using the parametric independent sample *t* test. Proportion was compared using the Fischer’s exact test. Assessment of correlation between variables was done using Pearson’s correlation. A probability value of <0.05 (two-tailed) was considered significant.

## Results

Five hundred adult patients were admitted to our ICU during the study period of 2 years. Out of these, 375, 225, and 150 were in sepsis, severe sepsis, and septic shock, respectively. We successfully conducted TTE in 59 % (88/150) of septic shock patients. Pre-existing heart disease, poor echocardiographic window, hemodynamic instability during optimum positioning, inability to perform TTE within first 24 h of ICU admission and refusal of consent were reasons for exclusion.

### Baseline clinical characteristics

The mean age of the study population (*N* = 88) was 38 ± 15 years with gender (M:F) distribution of 52:36. Frequency of medical illness (91 %; 80/88) in the decreasing order was as follows: the lung (52 %), renal (41 %), diabetes mellitus (23 %), and hypertension (11 %), respectively. Baseline clinical characteristic of all patients are as depicted in Table 1. Life-support therapies like vasopressors (100 %; 88/88), mechanical ventilation (~88 %; 76/88), and RRT (~16 %; 14/88) were instituted as appropriate. Mean length of ICU stay was 21 ± 21 days.

**Table 1:** Baseline clinical characteristics of survivors and non-survivors in the post-resuscitation phase of septic shock

Variables	Septic shock	Survivors	Non-survivors	*P* value
	(*N* = 88)	(*n* = 36)	(*n* = 52)	
Age, years	38 ± 15	34 ± 13	41 ± 16	0.00*
Gender, female, *n* (%)	36 (41)	14 (39)	22 (42)	0.83
Type of illness, *n* (%)				
Medical	80 (91)	35 (44)	45 (56)	0.75
Surgical	8 (9)	1 (12)	7 (88)	0.15
Nature of illness, *n* (%)				
Lung disease	46 (52)	14 (39)	32 (61)	0.05
Renal disease	36 (41)	12 (33)	24 (46)	0.27
Comorbid illness, *n* (%)	34 (39)	14 (41)	20 (59)	1.0
Diabetes mellitus	20 (23)	8 (40)	12 (60)	1.0
Hypertension	10 (11)	4 (40)	6 (60)	1.0
Severity scores				
APACHE-II	16.5 ± 7.1	14.6 ± 7.0	17.9 ± 6.9	0.02*
SOFA	10.2 ± 4.5	10.1 ± 4.2	10.3 ± 4.7	0.02*
Laboratory				
Hemoglobin, g/dl	10.2 ± 6.5	9.1 ± 2.2	10.9 ± 8.2	0.19
TLC, ×10^3^/μL	17.9 ± 9.5	19 ± 12.1	17.2 ± 7.2	0.06
Platelet, ×10^3^/μL	143 ± 132	160 ± 148	130 ± 97	0.13
Serum creatinine, mg/dl	5.5 ± 2.8	2.4 ± 2.5	7.6 ± 2.6	0.17
Blood urea nitrogen, mg/dl	54.8 ± 42.2	45.1 ± 28.3	61.3 ± 48.4	0.02*
Total bilirubin, mg/dl	4.0 ± 3.5	2.7 ± 1.4	5.3 ± 3.6	0.00*
ALT, U/L	142 ± 130	167 ± 102	124 ± 111	0.37
AST, U/L	160 ± 130	185.4 ± 106.8	141.9 ± 103.5	0.23
ALP, U/L	274 ± 100	353.6 ± 182.1	215.4 ± 136.4	0.47
Albumin, g/dl	2.9 ± 0.7	3.1 ± 0.9	2.7 ± 0.4	0.00*
Hemodynamics				
Heart rate, bpm	103 ± 20	100 ± 19	105 ± 22	0.07
MAP, mmHg	87 ± 9	89 ± 11	85 ± 8	<0.001**
CVP, mmHg	8 ± 4	8 ± 3	9 ± 4	0.00*
Central venous O_2_ saturation, %	73 ± 7	73 ± 6	73 ± 8	0.82
Lactate, mg/dl	18 ± 11	15.9 ± 9	18.9 ± 12.8	0.01*
Acid-base				
pH	7.36 ± 0.08	7.38 ± 0.05	7.35 ± 0.10	<0.001**
Base excess	2.4 ± 2.3	1.5 ± 1.3	3.2 ± 2.7	0.00*
Base deficit	4.1 ± 3.4	4.5 ± 3.5	3.9 ± 3.4	0.64
PaO_2_, mmHg	103 ± 26	104 ± 26	103 ± 26	0.02*
PaCO_2_, mmHg	42 ± 13	39 ± 7	44 ± 16	0.00*
cHCO_3_	23.6 ± 5	23 ± 4.1	24 ± 5.6	0.35
Na^+^, mEq/L	140 ± 8.8	139 ± 10	140 ± 7.8	0.25
K^+^, mEq/L	4.9 ± 1.6	5.5 ± 0.4	4.4 ± 0.5	0.36
Cl^−^, mEq/L	101 ± 6.4	100 ± 6.9	102 ± 6.1	0.57
Mechanical ventilation				
Invasive ventilation, *n* (%)	76 (88)	30 (43)	46 (57)	0.54
PaO_2_/FiO_2_ ratio	251 ± 95	254 ± 70	248 ± 109	<0.001**
Pressure support, cmH_2_O	17 ± 5	17 ± 3	17 ± 6	0.04*
PEEP, cmH_2_O	8 ± 3	9 ± 2	8 ± 3	0.45
Tidal volume, ml	420 ± 91	459 ± 63	395 ± 97	0.02*
Fluid balance				
Last 24 h, ml	1163 ± 940	1418 ± 1028	1009 ± 868	0.60
Positive balance, *n* (%)	60 (68)	20 (33)	40 (67)	0.19
Negative balance, *n* (%)	20 (23)	10 (50)	10 (50)	
Urine output				
Previous 24 h, ml	1461 ± 1058	1658 ± 987	1325 ± 1094	0.16
Unaided, *n* (%)	50 (57)	22 (44)	28 (56)	0.58
Lasix aided, *n* (%)	14 (16)	4 (29)	10 (71)	
Dialysis, *n* (%)	14 (16)	6 (43)	8 (57)	
Hemodynamic support				
Vasopressors, *n* (%)	88 (100)	36 (44)	52 (56)	0.16
Vasopressin, *n* (%)	28 (32)	6 (21)	22 (79)	0.02*
NADR dose, μg/kg/min	0.2 ± 0.1	0.10 ± 0.12	0.26 ± 0.26	<0.001**
NADR ≥0.1 μg/kg/min	44 (50)	8 (18)	36 (82)	<0.001**
Dobutamine, *n* (%)	6 (7)	4 (67)	2 (33)	0.22
Event free, days				
Ventilation	9 ± 8.5	15.7 ± 15	3.9 ± 6.3	<0.001**
Vasopressor	16.4 ± 15.6	31.4 ± 30.1	5.2 ± 3.7	<0.001**
RRT	21.1 ± 17	32.5 ± 30.5	12.6 ± 11.2	0.06
Length of ICU stay, days	21 ± 21	33.4 ± 30.9	12.1 ± 9.3	0.04*

All measurements are in mean ± SD, unless specified

*Abbreviations*: *APACHE-II* acute physiology and chronic health evaluation, *SOFA* sequential organ failure assessment, *ALT* alanine transaminase, *AST* aspartate aminotransferase, *ALP* alkaline phosphatase, *MAP* mean arterial pressure, *CVP* central venous pressure, *PaO*_*2*_ partial pressure of oxygen, *K* ^*+*^ potassium, *Cl* ^*−*^ chloride, *PEEP* positive end-expiratory pressure, *NADR* noradrenaline, *RRT* renal replacement therapy, *ICU* intensive care unit

*Significant, *p* < 0.05; **highly significant, *p* < 0.001

### Comparison between survivors and non-survivors

Forty-one percent (36/88) of patients survived. Baseline clinical characteristics of survivors (Ss) and non-survivors (NSs) are as depicted in Table 1. Significant difference was observed in the following: age, severity scores (APACHE-II and SOFA), BUN, bilirubin, albumin, MAP, CVP, lactate, pH, base excess, PaO_2_, PaCO_2_, PaO_2_/FiO_2_ ratio, pressure support, tidal volume, hemodynamic support variables, event (ventilation, vasopressor) free days, and length of ICU stay.

### Right heart echocardiographic parameters

**Fig. 1:**
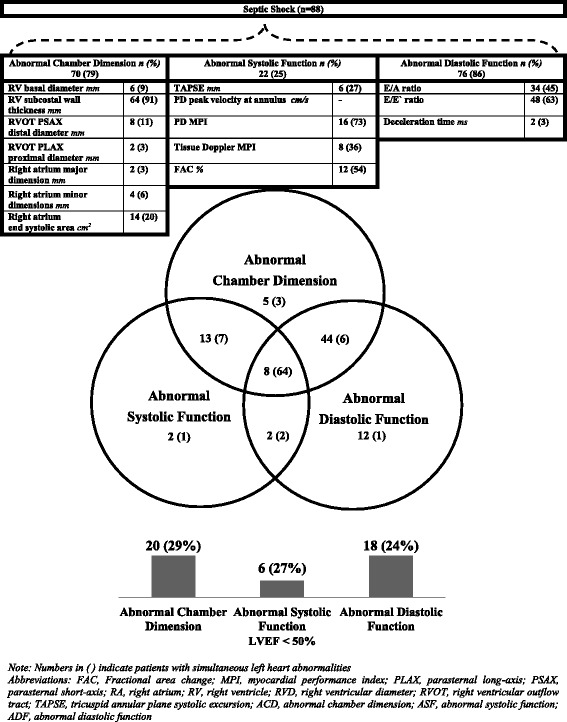
Frequency and distribution of right heart abnormalities in septic shock. *Numbers in parentheses* indicate patients with simultaneous *left* heart abnormalities

Seventy (79 %, 70/88) patients with septic shock had abnormal chamber dimension; while 25 and 86 % had abnormal systolic and diastolic function, respectively, in our study. The proportion of these abnormalities and their respective distribution alone or in various combinations was as represented in the Venn diagram of Fig. 1. Also depicted in Fig. 1 is a bar graph showing the number of patients with simultaneous LV systolic dysfunction (defined as LVEF < 50 % in this study). Tables 2 and 3 depict right and left heart TTE indices of the study population respectively.

**Table 2:** Dimensional and functional echocardiographic parameters of the right heart in septic shock

Dimensional and functional indices of right heart, mean ± SD	Normal non-indexed population reference limits [15]	Total septic shock (*N* = 88)	Septic shock		*P* value	95 % CI of the difference
			Survivor	Non-survivor		
			(*n* = 36)	(*n* = 52)		
Chamber dimensions						
RV basal diameter, mm	≤42	33.12 ± 6.66	32.26 ± 5.03	33.75 ± 7.61	0.41	−03.00 to +01.30
RV SC WT, mm	≤5	6.85 ± 1.56	6.71 ± 1.77	6.95 ± 1.42	0.96	−00.80 to +00.75
RVOT-PSAX distal diameter, mm	≤27	23.77 ± 4.61	23.71 ± 3.22	23.82 ± 5.35	0.42	−00.74 to +01.80
RVOT-PLAX proximal diameter, mm	≤33	24.23 ± 4.98	23.71 ± 3.48	24.57 ± 5.76	0.82	−01.30 to +01.60
RA major dimension, mm	≤53	33.33 ± 8.98	32.67 ± 6.06	33.79 ± 10.57	0.98	−02.70 to +02.70
RA minor dimensions, mm	≤44	26.73 ± 8.74	25.97 ± 8.56	27.26 ± 8.91	0.28	−01.10 to +03.70
RA end-systolic area, cm^2^	≤18	16.40 ± 17.05	20.79 ± 25.31	13.23 ± 4.74	0.80	−06.10 to +04.70
Systolic functions						
TAPSE, mm	≥16	23.29 ± 5.84	23.56 ± 4.45	23.10 ± 6.67	0.47	−01.10 to +02.40
PD peak velocity at annulus, cm/s	≥10	71.19 ± 22.41	71.50 ± 22.45	70.97 ± 22.62	0.86	−07.00 to +05.90
PD-MPI	≤0.40	0.81 ± 2.81	0.36 ± 0.08	1.12 ± 3.64	0.16	−01.20 to +00.21
TD-MPI	≤0.55	0.39 ± 0.13	0.40 ± 0.13	0.39 ± 0.13	0.33	−00.07 to +00.20
FAC, %	≥35	42.10 ± 8.92	43.25 ± 6.26	41.36 ± 10.26	0.04	00.08 to +06.40
Diastolic functions						
E/A ratio	0.8–2.1	1.34 ± 0.64	1.21 ± 0.43	1.43 ± 0.75	0.22	−00.30 to +00.07
E/E′ ratio	≤6	7.75 ± 4.14	7.00 ± 3.67	8.30 ± 4.42	0.17	−02.20 to +00.40
Deceleration time, ms	≥120	164.88 ± 29.22	169.44 ± 26.29	161.60 ± 30.99	0.50	−06.60 to +13.40

*Abbreviations*: *RV* right ventricle, *RV SC WT* RV subcostal wall thickness, *RVOT* right ventricular outflow tract, *PLAX* parasternal short-axis, *PSAX* parasternal long-axis, *RA* right atrium, *TAPSE* tricuspid annular plane systolic excursion, *PD* pulse Doppler, *TD* tissue Doppler, *MPI* myocardial performance index, *FAC* fractional area change

**Table 3:** Dimensional and functional echocardiographic parameters of the left heart in septic shock

Dimensional and functional indices of the right heart, mean ± SD	Normal non-indexed population reference limits [15]	Total septic shock (*N* = 88)	Septic shock		*P* value	95 % CI of the difference
			Survivor	Non-survivor		
			(*n* = 36)	(*n* = 52)		
Chamber dimensions						
RV basal diameter, mm	≤42	33.12 ± 6.66	32.26 ± 5.03	33.75 ± 7.61	0.41	−03.00 to +01.30
RV SC WT, mm	≤5	6.85 ± 1.56	6.71 ± 1.77	6.95 ± 1.42	0.96	−00.80 to +00.75
RVOT-PSAX distal diameter, mm	≤27	23.77 ± 4.61	23.71 ± 3.22	23.82 ± 5.35	0.42	−00.74 to +01.80
RVOT-PLAX proximal diameter, mm	≤33	24.23 ± 4.98	23.71 ± 3.48	24.57 ± 5.76	0.82	−01.30 to +01.60
RA major dimension, mm	≤53	33.33 ± 8.98	32.67 ± 6.06	33.79 ± 10.57	0.98	−02.70 to +02.70
RA minor dimensions, mm	≤44	26.73 ± 8.74	25.97 ± 8.56	27.26 ± 8.91	0.28	−01.10 to +03.70
RA end-systolic area, cm^2^	≤18	16.40 ± 17.05	20.79 ± 25.31	13.23 ± 4.74	0.80	−06.10 to +04.70
Systolic functions						
TAPSE, mm	≥16	23.29 ± 5.84	23.56 ± 4.45	23.10 ± 6.67	0.47	−01.10 to +02.40
PD peak velocity at annulus, cm/s	≥10	71.19 ± 22.41	71.50 ± 22.45	70.97 ± 22.62	0.86	−07.00 to +05.90
PD-MPI	≤0.40	0.81 ± 2.81	0.36 ± 0.08	1.12 ± 3.64	0.16	−01.20 to +00.21
TD-MPI	≤0.55	0.39 ± 0.13	0.40 ± 0.13	0.39 ± 0.13	0.33	−00.07 to +00.20
FAC, %	≥35	42.10 ± 8.92	43.25 ± 6.26	41.36 ± 10.26	0.04	00.08 to +06.40
Diastolic functions						
E/A ratio	0.8–2.1	1.34 ± 0.64	1.21 ± 0.43	1.43 ± 0.75	0.22	−00.30 to +00.07
E/E′ ratio	≤6	7.75 ± 4.14	7.00 ± 3.67	8.30 ± 4.42	0.17	−02.20 to +00.40
Deceleration time, ms	≥120	164.88 ± 29.22	169.44 ± 26.29	161.60 ± 30.99	0.50	−06.60 to +13.40

*Abbreviations*: *LV* left ventricle, *D* diastole, *S* systole, *LVWT-IVS* LV wall thickness-inter ventricular septum, *PW* posterior wall, *LA* left atrium, *PD* pulse Doppler, *MPI* myocardial performance index, *TD* tissue Doppler, *LVEF* LV ejection fraction

### Comparison between survivors and non-survivors

#### Chamber dimensions

In decreasing order, the proportion of abnormal chamber dimensions were as follows: RV subcostal wall thickness ~91 %; RA ESA ~20 %; right ventricular outflow tract (RVOT)-PSAX distal diameter ~11 %; RV basal diameter ~9 %; RA minor dimension ~6 %; and RA major dimension and RVOT-PLAX proximal diameter ~3 % each. All indices of chamber dimensions were not significantly different between Ss and NSs (Table 2).

### Systolic function

Approximately 25 % (22/88) of patients with septic shock had abnormal systolic function. In decreasing order, the proportion of these abnormalities were as follows: PD-MPI ~73 %; FAC ~54 %; TD-MPI ~36 %; and TAPSE (mm) ~27 %. Except for FAC (*p* = 0.04), comparison of systolic function indices between Ss and NSs (Table 2) was non-significant.

### Diastolic function

Approximately 86 % (76/88) of patients with septic shock had abnormal diastolic function. In decreasing order, the proportion of abnormal RV diastolic function indices were as follows: E/E′ ratio ~63 %; E/A ratio ~45 %; and deceleration time ~3 %. The comparisons of the above diastolic function indices between Ss and NSs amongst septic shock (Table 2) were non-significant.

### Distribution of right heart abnormalities

RH with isolated abnormal chamber dimension and systolic and diastolic functions was present in 5, 2, and 12 patients, respectively. A maximum of 44 patients had both chamber and diastolic function abnormalities, while 8 patients had all three (chamber and systolic/diastolic) abnormalities. All patients with three RH abnormal indices also had simultaneously similar LH abnormalities. However, similar distribution was not observed vice versa (Fig. 1).

### Correlation between right and left heart abnormalities

**Fig. 2:**
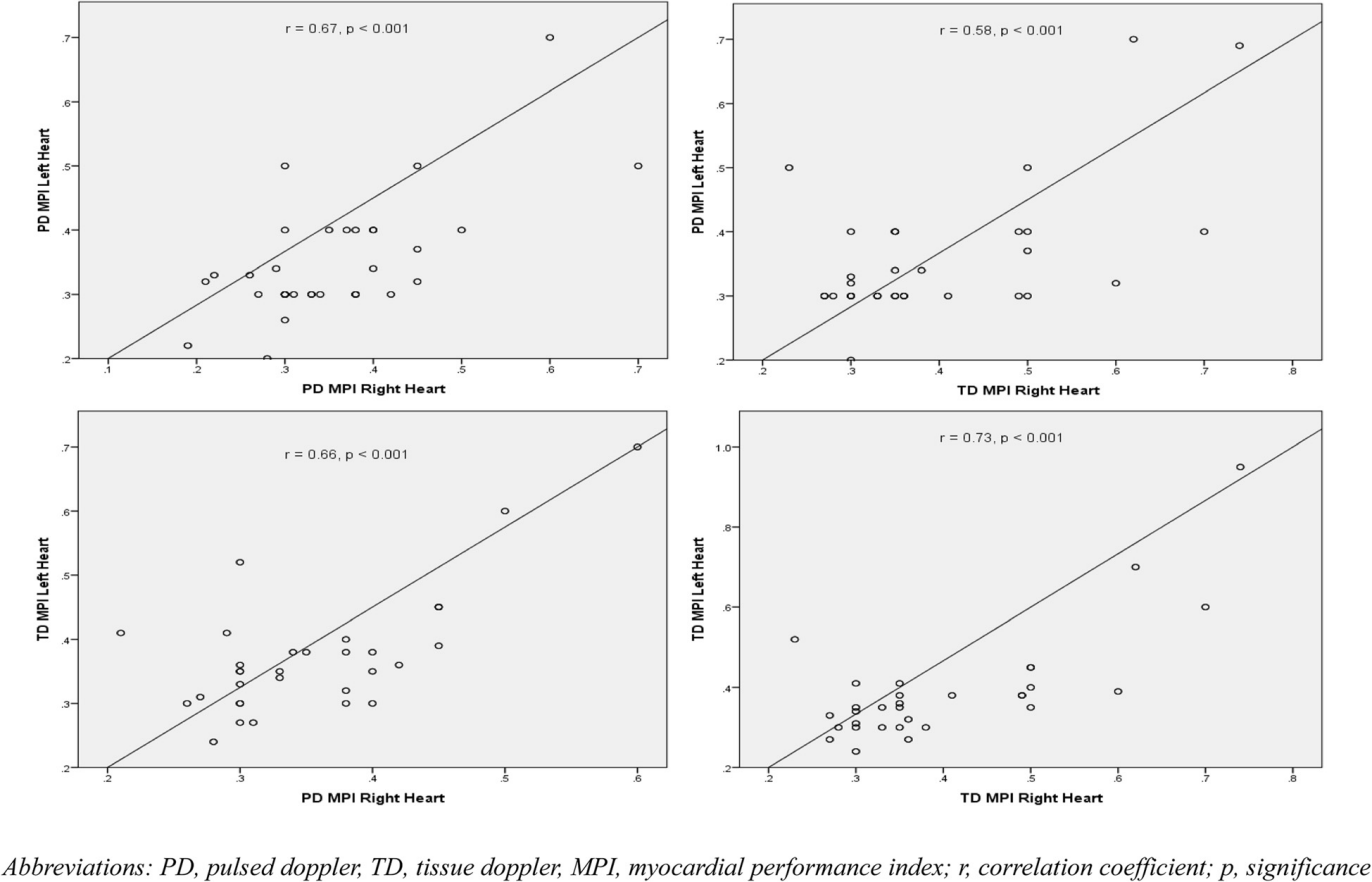
Correlation between myocardial performance index of the right and left heart

The incidence of LV systolic dysfunction was present in approximately 23 % (20/88) of septic shock patients. Within these, 20, 6, and 18 had RH abnormal chamber, systolic, and diastolic functions, respectively. Significant correlation was observed between the PD and TD-MPI of the right and left heart (Fig. 2).

### Life-support therapies and right heart echocardiographic indices

#### Ventilation

A comparative analysis (table not shown) of ventilated (*n* = 76) and non-ventilated (*n* = 12) patients for RH TTE indices was not significantly different. Ventilated patients had a significantly higher noradrenaline dose (*p* < 0.001) and urine output (*p* = 0.007).

#### Vasopressors

Based on noradrenaline dose of ≥0.1 μg/kg/min, patients of septic shock were re-categorized (table not shown). There were 44 patients who required ≥0.1 μg/kg/min dose. Comparative analysis of RH TTE indices in patients with (*n* = 44) and without (*n* = 44) this dose differed significantly for FAC (with, 39.67 ± 10.41 vs. without, 38.44 ± 6.4; *p* = 0.01). At higher doses ≥0.3 μg/kg/min (*n* = 22), further significant reduction was observed in FAC (*p* < 0.001).

#### Fluids

Patients with positive (*n* = 58) and negative (*n* = 20) fluid balance (fb) (ml) in the previous 24 h of TTE were compared (table not shown). Significant differences were observed in the following RH indices: RA minor dimension (positive fb, 27.9 ± 9.9 vs. negative fb, 24.7 ± 3.9; *p* = 0.039) and TD-MPI (positive fb, 0.39 ± 0.12 vs. negative fb, 0.34 ± 0.03; *p* = 0.024).

## Discussion

Myocardial depression though well recognized in septic patients is still poorly understood. Its impact on outcome still remains debatable [16]. However, despite RH being equally involved [17], it has been discussed minimally in septic shock [7, 8, 18–21]. As of now, no comprehensive baseline data of RH in septic shock exists. Furthermore, there is confusion regarding which single or constellation of components (chamber dimension, systolic, and diastolic function) defines acute RH dysfunction [15]. In this context, the present exploratory study evaluated the impact of RH function on management and outcomes in septic shock. To accomplish this, we performed a comprehensive RH TTE in the post-resuscitation phase of septic shock within the first 24 h of ICU admission as per established ASE guidelines [15]. The main findings from our study were that RH abnormalities (dimensional and functional) exist in high proportion in septic shock patients. Subcostal wall thickness, PD-MPI, and E/E′ are the predominant abnormalities in chamber dimension and systolic and diastolic functions of RV, respectively. However, apparent differences between Ss and NSs were not statistically significant, except for FAC.

Various methods like radionuclide [18], pulmonary artery catheter [19–21], and TTE [7, 8] have been used for evaluating the RH. Furthermore, various indices have been used to describe the dimensional and functional aspects of the RH. Included amongst these are ejection fraction [16–19], stroke volume change [8] for RV function and end-diastolic volume index [16, 19], diameter [7], and end-diastolic area [8] for RV dimension. However, their clinical significance remains questionable [15]. Incidence of RH dysfunction in septic shock is difficult to estimate in the absence of a well-defined universally accepted definition. Therefore, we attempted to describe indices of dimension and function as either normal or abnormal in comparison to reference values [15].

Discriminative power of RH indices (except FAC) was not observed in our study. Furian T et al. [7] described the presence of RV dysfunction (defined by TD peak systolic velocity [RV-Sm] of <12 cm/s) in severe sepsis as discriminative between Ss and NSs. Landesberg G et al. [8] in their study on diastolic dysfunction utilized RV areas (end-diastolic and -systolic) for the assessment of RV function in severe sepsis and septic shock and did not find them differentiating Ss from NSs. In another study by Landesberg G et al. [22] in 106 patients of severe sepsis and septic shock, authors concluded that RV systolic dysfunction could explain the association of troponin with mortality. Using thermodilution technique in septic shock patients, Vincent JL et al. [20, 21] observed that NSs had lower RVEF. Our study describes both dimensional and functional aspects of the RH in complete accordance to ASE guidelines, as against previous investigators who relied on fewer and lesser standardized indices. Our results are similar to Landseberg G et al. [8] but dissimilar to studies by Furian et al. [7], Vincent et al. [20, 21], and Landesberg G et al. [22]. The issue whether early ventricular dysfunction or dilatation decreases mortality in adults with severe sepsis and septic shock was also explored in a meta-analysis [14]. Huang et al. [14] included more than 700 and 400 patients of the left and right ventricle dysfunction/indices, respectively, and concluded that there was no statistical difference in RV dimension or function between Ss and NSs. The findings from our study are in conformity with this meta-analysis and notably are even more comprehensive as they include many newer indices like TAPSE and PD/TD-MPI of RH.

Correlation between many indices of the right and left heart was an expected observation. We have only depicted those with maximum significance. Septic shock patients invariably require life-support therapies like invasive ventilation, vasopressors, and fluids. Understanding the complex interaction between these factors, either individually or in combination, along with disease per se on RH is challenging. Understandably, previous studies have only minimally explored this issue [14]. Ventilation increases RH afterload due to increase in pulmonary vascular resistance. In our study, ventilated patients showed increased chamber dimensions, and reduced systolic and diastolic functions of RH, though non-significantly. However, interpretation needs to be cautious as majority of our patients were ventilated. Vasopressors inevitably have dose-dependent direct effects on the pulmonary circulation and myocardium. Noradrenaline increases pulmonary vascular resistance but does not increase RVEF [23]. In our study, FAC decreased with increasing dose of noradrenaline, probably as a result of dose-dependent increase in pulmonary vascular resistance. However, any future study on the above issues also needs to analyze the alterations in RH at various levels, duration, and timing of vasopressors. Patients with positive fb had higher MPI emphasizing the fact that the RH is preload dependent. Most studies of RH echocardiography in septic shock discuss minimally about the timing of fluid administration, effect of vasopressors, and mechanical ventilation [7, 8, 18–21].

### Limitations

Preexisting diseases in patients of septic shock, limited sample size from a single institution, and single snaps taken in the entire course of septic shock when its onset is clearly not defined are significant limitations of our study. Worst cardiac indices may have been missed in our study despite TTE being performed on the very first day of ICU admission and apparently also the first day of septic shock. This is reflected by stable vitals and low APACHE-II scores in the relatively more stable post-resuscitation phase of septic shock. Many of the TTE parameters of RV recorded in this study are difficult to obtain accurately due to problems of either angulation (e.g., E and E′ velocities of the TV) or irregular shape of RV (e.g., ESA, which depends strongly on the level at which they are measured) and very thin thickness of RV. Furthermore, in the absence of repeat TTE, potential reversibility of the RH abnormalities could also not be established. Indexed RH indices are desirable but could not be performed due to weight-measurement-related issues in our ICU. The absence of provision of TEE in our study resulted in loss of data in cases wherein TTE could not be performed due to reasons previously mentioned. Comparison of TTE cardiac parameters of ICU patients with normal non-ICU population may seem unjustified. However, non-existence of detailed baseline indices as suggested by ASE from ICU patients makes it a reasonable alternative as of now. Multiple comorbidities in our study population with underlying pulmonary, renal, hypertension, and diabetes may not render our findings generalizable to all populations. The present exploratory study is only a humble attempt to better understand RH in septic shock utilizing universally accepted ASE guidelines. The complex interplay of multifactorial aspects of positive intrathoracic pressure, right atrial pressure, mean systemic filling pressure, RV/LV cardiac output, pulmonary/systemic vascular resistance, hypoxic pulmonary vasoconstriction, and RV/LV interdependence in patients of septic shock on vasopressors is too complicated to comprehend from our study with abovementioned limitations.

## Conclusions

ASE approach to RH assessment allows for better echocardiographic evaluation. Though RH abnormalities exist in high proportions in patients of septic shock, they did not predict mortality in our study. While feasibility of performing TTE for RH function in all septic shock patients is arguable, efforts should continue to gather more data from disease-specific patient population. Future studies should focus on indexing and grading the severity of RH abnormalities to better objectively classify RH dysfunction. Future studies with this basic knowledge may also pave the pathway to better understand complex heart-lung interactions in positive-pressure-ventilated patients of septic shock on vasopressors.

## Abbreviations

ALP: alkaline phosphatase
ALT: alanine transaminase
APACHE-II: acute physiology and chronic health evaluation
AST: aspartate aminotransferase
Cl^−^: chloride
CVP: central venous pressure
FAC: fractional area change
ICU: intensive care unit
K^+^: potassium
MAP: mean arterial pressure
MPI: myocardial performance index
NADR: noradrenaline
NSs: Non-survivors
PaO_2_: partial pressure of oxygen
PEEP: positive end-expiratory pressure
PLAX: parasternal long-axis
PSAX: parasternal short-axis
RA: right atrium
RRT: renal replacement therapy
RV: right ventricle
RVOT: right ventricular outflow tract
Ss: Survivors
SOFA: sequential organ failure assessment
TAPSE: tricuspid annular plane systolic excursion
